# Cardiac Rehabilitation in Patients After Coronary Artery Bypass Grafting: Core Components and Long-Term Follow-Up

**DOI:** 10.3390/jcm15031103

**Published:** 2026-01-30

**Authors:** Irina Prisacariu, Luana-Viviana Iorescu, Chaimae Aboueddahab, Maryam Taheri, Eirini Beneki, Buket Akinci, Ladislav Batalik, Silviu Ionel Dumitrescu, Maria Marketou, Francesco Perone

**Affiliations:** 1 Central Military Emergency University Hospital “Dr. Carol Davila”, 010825 Bucharest, Romania; irina.prisacariu96@gmail.com (I.P.); luanaiorescu@yahoo.com (L.-V.I.); silviudumius@yahoo.com (S.I.D.); 2 Department of Cardiology, Ibn Sina University Hospital, Mohammed V University of Rabat, Rabat 10000, Morocco; ch.aboueddahab@gmail.com; 3 Department of Cardiovascular Medicine, Mayo Clinic, Rochester, MN 55905, USA; dr.maryam.taheri@outlook.com; 4 Department of Cardiology, CHUV Lausanne University Hospital, 1005 Lausanne, Switzerland; e.beneki@hotmail.com; 5 Department of Physiotherapy and Rehabilitation, Faculty of Health Sciences, Biruni University, 34010 Istanbul, Turkey; barbuket@hotmail.com; 6 Biruni University Research Center (B@MER), Biruni University, 34010 Istanbul, Turkey; 7 Department of Rehabilitation, University Hospital Brno, 625 00 Brno, Czech Republic; batalik.ladislav@fnbrno.cz; 8 Department of Physiotherapy and Rehabilitation, Faculty of Medicine, Masaryk University, 602 00 Brno, Czech Republic; 9 Rehabilitation Clinic, Faculty of Medicine, Masaryk University, 625 00 Brno, Czech Republic; 10 Department of Public Health, Faculty of Medicine, Masaryk University, 625 00 Brno, Czech Republic; 11 School of Medicine, University of Crete, 71110 Crete, Greece; maryemarke@yahoo.gr; 12 Cardiac Rehabilitation Unit, Rehabilitation Clinic “Villa delle Magnolie”, 81020 Castel Morrone, Caserta, Italy

**Keywords:** cardiac rehabilitation, coronary artery bypass grafting, coronary artery disease, secondary prevention, aerobic exercise training, resistance exercise training, physical activity

## Abstract

Cardiac rehabilitation is strongly recommended in secondary cardiovascular prevention. In patients after coronary artery bypass grafting, this intervention is suggested to reduce mortality, morbidity, and disability. In addition, rehabilitation programs improve quality of life and cardiorespiratory fitness. Modern cardiac rehabilitation programs include structured exercise training, education, nutritional counseling, psychosocial support, and management of cardiovascular risk factors, each tailored to the specific needs of post-coronary artery bypass grafting patients who often face a high burden of comorbidities and surgical recovery challenges. For these reasons, cardiac rehabilitation should be regarded as standard of care. Evidence supports early cardiac rehabilitation initiation and individualized multidisciplinary plans, which have shown to improve exercise capacity, health-related quality of life, and medication adherence. Long-term follow-up is essential, as studies have demonstrated a clear association between sustained cardiac rehabilitation engagement and decreased rates of rehospitalization and all-cause mortality. Therefore, this comprehensive review presents recent advances and updates on the management of patients after coronary artery bypass grafting during cardiac rehabilitation, with a focus on the core components and long-term follow-up.

## 1. Introduction

Coronary artery disease (CAD) remains the leading cause of global mortality [1], and coronary artery bypass grafting (CABG) remains one of the most common and effective surgical interventions for the treatment of advanced CAD, particularly in patients with multivessel disease or left main coronary artery involvement. Despite its success in improving myocardial perfusion and reducing anginal symptoms, CABG does not eliminate the underlying atherosclerotic process [2]. Without comprehensive secondary prevention strategies, including lifestyle modifications and optimized medical therapy, patients remain at elevated risk for recurrent cardiovascular events, hospitalizations, and diminished quality of life [3,4,5].

Widely endorsed by major international guidelines—including those from the American Heart Association (AHA), American College of Cardiology (ACC), and European Society of Cardiology (ESC)—cardiac rehabilitation (CR) has become a central pillar of secondary prevention, offering structured, multidisciplinary interventions for post-CABG patients that integrate supervised exercise, nutritional counseling, psychosocial support, smoking cessation, and comprehensive risk factor management; compelling evidence confirms that CR participation substantially reduces mortality, rehospitalization rates, and enhances both physical function and psychological well-being [6,7,8].

The physiological impact of exercise-based CR is particularly pivotal in the post-CABG population, where structured aerobic and resistance training enhances cardiopulmonary capacity by elevating peak oxygen uptake, improving autonomic regulation, and facilitating myocardial oxygen delivery when initiated early and maintained consistently [9]. In parallel, CR significantly reduces major adverse cardiovascular events and readmission rates, while promoting comprehensive gains in health-related quality of life—including physical function, emotional well-being, and overall vitality—thus reinforcing its role as a prognostic modifier in post-surgical recovery [10]. Additionally, evidence suggests that individualized rehabilitation interventions can beneficially modulate systemic inflammatory responses and endothelial function, further diminishing the risk of postoperative complications and long-term cardiovascular morbidity [11].

Recent data from the multinational INTERASPIRE study reveal a global failure in secondary prevention: across 14 countries, only 9% of coronary heart disease patients attended most CR sessions, and just 1% met full guideline targets within a year. High rates of uncontrolled low-density lipoprotein cholesterol (LDL-C, 83%), blood pressure (61%), and dysglycaemia (67%) persisted, especially among women, who were less likely to reach treatment goals [4].

Despite its proven efficacy and inclusion in global guidelines, CR remains markedly underutilized, with significant disparities in access—particularly in low-and middle-income countries (LMICs), where only a fraction of eligible patients can participate due to limited program availability [12]. Barriers such as insufficient referral practices, lack of awareness, transportation, lack of reimbursement and other financial barriers, and geographic inaccessibility, especially in rural areas, continue to hinder enrollment. In response, home-based and tele-rehabilitation models have gained momentum, offering flexible, scalable, and cost-effective alternatives that can extend the reach of CR to underserved populations without compromising quality of care [12,13,14]. In CABG patients, the importance of CR extends beyond the immediate postoperative period. Long-term follow-up and risk factor management are vital to preserving surgical benefits, preventing graft failure, and reducing cardiovascular events. Sustained adherence to medical therapy, lifestyle changes, and exercise is essential for lasting improvements in prognosis [15].

In this comprehensive narrative review, we synthesize current evidence and recent updates on cardiac rehabilitation in patients after CABG, focusing on core components, physiological benefits, and long-term strategies to optimize recovery and prognosis. We aim to support clinicians in implementing phase-specific and personalized rehabilitation pathways for this high-risk population.

## 2. Post-Operative Period

Early, structured Phase I cardiac rehabilitation begins in the hospital and combines progressive mobilization, breathing exercises, and patient education to stabilize patients medically and prepare a seamless handoff to phase II CR [6,16,17,18]. Within Phase I, “early mobilization” has been variably defined; two studies offered a clear operational definition: gradual activity starting postoperative day 1 with independent ambulation by day 5 [19,20]. In the systematic review by Ramos dos Santos et al., first-day activities commonly included body positioning, sitting at the edge of the bed, passive and active limb exercises, and assisted ambulation; several trials also incorporated respiratory techniques such as incentive spirometry, deep-breathing, and intermittent positive pressure. However, the exact content and start time varied between protocols. Timing ranged from during intubation to post-extubation or the first postoperative day, and up to 48–72 h after extubation across studies, underscoring that early mobilization is a continuum of care rather than a single maneuver [21]. Moradian et al. started graded mobilization 2 h after extubation and observed higher PaO_2_/SaO_2_ and lower rates of atelectasis and pleural effusion versus usual care after CABG [22]. Meta-analyses confined to cardiac surgery further show that early mobilization improves functional capacity at discharge (e.g., six-minute walk distance by ~+54 m, 95% CI 31–77) and may shorten hospitalization without excess adverse events [23,24]. Furthermore, the respiratory physiotherapy delivered in Phase I typically includes coached deep breathing, supported cough/huff, early ambulation, and, selectively, devices such as incentive spirometry (IS) or positive expiratory pressure. A trial of deep breathing reduced postoperative atelectasis after CABG [25]. A randomized trial showed that higher-frequency deep-breathing exercises with positive expiratory pressure (PEP) significantly improved early postoperative oxygenation after cardiac surgery [26]. Yazdannik et al. conducted a randomized clinical trial showing that incentive spirometry improved arterial blood gas parameters in post-CABG patients [27]. Whereas Renault et al. found no superiority of IS over deep breathing for spirometry and oxygenation after CABG [28]. In line with the latter, a recent cardiac surgery–focused meta-analysis revealed that incentive spirometry was not superior to standard respiratory care for preventing postoperative pulmonary complications or improving clinical outcomes [29]. Therefore, modality selection can be individualized on a case-by-case basis and aligned with local expertise and resources (Table 1).

Before starting cardiac rehabilitation and early mobilization, baseline assessment of left ventricular (LV) function is a critical first step to ensure that these interventions are appropriate and beneficial. [30]. Traditional parameters include LV ejection fraction (LVEF) and global longitudinal strain (GLS) derived from speckle-tracking echocardiography (STE); LVEF is a useful screening tool but may underestimate subclinical dysfunction, whereas STE-GLS can reveal subclinical LV impairment even when LVEF is preserved. However, both indices are highly dependent on loading conditions, potentially limiting their diagnostic accuracy, particularly in patients with preserved ejection fraction or variable afterload [31]. Myocardial work (MW), a novel echocardiographic method that integrates LV deformation with afterload estimation via brachial cuff blood pressure, offers a more load-adjusted assessment of LV performance [31,32]. MW analysis yields four indices—global work index (GWI), global constructive work (GCW), global wasted work (GWW), and global work efficiency (GWE)—each providing complementary insights into myocardial mechanics [31,32]. Evidence supports MW’s utility across various cardiovascular conditions, including acute coronary syndromes, valvular heart disease, hypertension, and cardiomyopathies [31,33]. In a recent prospective observational study of post-CABG patients with preserved LVEF, Perone et al. demonstrated significant improvements in GWI and GCW following a structured CR program, alongside enhanced exercise capacity on the six-minute walk test [34]. These findings suggest MW parameters can sensitively track functional recovery and training-induced myocardial adaptations in this population. Furthermore, prior studies in acute coronary syndrome patients have shown that MW can detect subclinical systolic dysfunction and capture the impact of exercise-based rehabilitation [31,35].

**Table 1:** Overview of studies focused on Phase I CR after CABG.

Study (First Author, Year)	Design & Population	Start Timing	Phase I Components	Comparator	Primary Outcomes	Key Findings
Herdy, 2008 [36]	RCT; hospitalized CABG patients, n = 56 patients	Pre-operative program resumed post-extubation to discharge	Pre-/post-operative cardiopulmonary rehabilitation includes progressive mobilization and respiratory exercises	Usual care	Extubation time; ward length of stay; complications; 6MWT; peak flow	Shorter time to extubation (*p* = 0.05) and ward stay (*p* < 0.001); fewer complications in intervention
Moradian, 2017 [22]	Randomized clinical trial in post-CABG, n = 100 patients	≈2 h after extubation (graded progression)	Early graded mobilisation under monitoring	Usual care	Oxygenation; atelectasis; pleural effusion	Better oxygenation and fewer pulmonary complications in intervention group
Cassina, 2016 [37]	Observational ICU pilot after cardiac surgery, n = 53 patients	Early postop when hemodynamically stable; continuous monitoring	Supervised early mobilisation in ICU	— (safety/feasibility)	Hemodynamic tolerance; safety	Feasible and safe with stability + monitoring
Borzou, 2018 [38]	Randomized trial, n = 60 patients	≈72 h after surgery to discharge	Structured education + simple exercises (breathing; limb movements; bedside/chair), group + 1:1	Routine care	Self-efficacy at discharge and 1 month	Higher self-efficacy vs. control (all *p* < 0.001)

6MWT, six minute walking test; CABG, coronary artery bypass grafting; CR, Cardiac rehabilitation; ICU, intensive care unit; RCT, Randomized Controlled Trial.

## 3. Phase II Cardiac Rehabilitation After CABG: Initial Assessment, Exercise Training, and the Core Components

Phase II CR is a critical window in the recovery process and it is focused on restoring physical function, enhancing psychological well-being and reducing cardiovascular risk through comprehensive, multidisciplinary interventions. At the core of this phase lies the initial assessment, which is essential for developing a safe and personalized exercise prescription and for identifying barriers to participation and adherence. The initial assessment includes symptom status, comorbidities, traditional and non-traditional cardiovascular risk factors, medication use, ECG, blood testing, transthoracic echocardiogram, a review of surgical outcomes, wound healing, and psychosocial risk factors [16,30,39]. A thorough functional capacity evaluation is crucial for tailoring exercise safely. Ideally, this involves a cardiopulmonary exercise test (CPET) to determine maximal oxygen uptake (VO_2_ peak) and ventilatory thresholds. If CPET is unavailable, symptom-limited exercise testing is suggested and, if the patient is unable to perform this, the 6-min walk test (6MWT) should be considered. These tests are also useful for monitoring other important parameters for exercise safety, such as heart rate, blood pressure, and exercise-induced symptoms, especially in this postoperative population [40].

Exercise prescription should follow the FITT model: Frequency, Intensity, Time, and Type of exercise. Specifically, aerobic training is preferably recommended 6–7 days per week, at moderate or moderate-to-high intensity. Each session should initially last 20–30 min and gradually increase to 45–60 min. The type of aerobic activity commonly includes treadmill walking, stationary cycling, or rowing. The training progression should be individualized, with gradual increases in duration and intensity based on tolerance and the absence of limiting symptoms or complications [9].

In addition to patient assessment and exercise training, the core components include:

1. Physical activity counselling (primarily assessing the type and level of activity);
2. Risk factor management (e.g., control of blood pressure with values < 130/80 mmHg, lipids with LDL-C < 55 mg/dL, glucose, and smoking cessation);
3. Nutritional counseling, typically based on the Mediterranean or DASH diet;
4. Psychosocial support, including depression and anxiety screening (integrating a structured intervention on psychosocial risk factors);
5. Medication adherence optimization;
6. Patient education on symptom recognition (such as a new episode of angina or an equivalent), lifestyle modification, and long-term self-care.

Exercise in CR also includes a resistance training component, introduced with attention to the recent sternotomy. Resistance training improves muscular strength, endurance, and metabolic profile. According to current recommendations, training begins at 30–70% of 1-repetition maximum (1-RM) for upper limbs and 40–80% for lower limbs, using resistance bands, light free weights (e.g., 1–5 kg), or machines. Sessions typically involve 1–3 sets of 12–15 repetitions, performed 2–3 times per week. It is essential to avoid exercises and the Valsalva maneuver which can provoke hemodynamic instability [41]. The combination of aerobic and resistance training has been shown to produce superior improvements in functional capacity and overall quality of life compared to aerobic training alone.

Safety monitoring during supervised sessions includes continuous or intermittent ECG, symptom checks, and hemodynamic parameters. High-risk patients (e.g., LVEF < 40%, recent arrhythmias) may require closer surveillance and lower training thresholds [42]. For long-term sustainability, patients are gradually transitioned to home-based or community-based programs, with periodic follow-up and remote support where possible. Recent evidence supports the implementation of hybrid and tele-rehabilitation programs, which integrate digital tools (apps, video sessions, and heart rate monitors) to extend the benefits of CR to underserved populations. Meta-analyses and multicenter trials have demonstrated that these models can safely match the effectiveness of center-based programs while improving accessibility and adherence, particularly in older or rural patients [14,43].

In summary, Phase II cardiac rehabilitation after CABG is a multifaceted intervention requiring careful initial assessment, individualized exercise prescription based on FITT principles, and the integration of key behavioral and clinical components. When delivered comprehensively and consistently, CR leads to substantial improvements in exercise capacity, mental health, and long-term cardiovascular outcomes.

## 4. Long-Term Follow-Up

Long-term management of patients after CABG plays a pivotal role in ensuring sustained clinical benefits, minimizing recurrent cardiovascular events, and improving overall quality of life. While early cardiac rehabilitation phases focus on inpatient and supervised outpatient programs, the continuation and expansion of these efforts into phase III and beyond are essential for maintaining functional capacity and adherence to treatment. Phase III cardiac rehabilitation primarily involves home-based and telerehabilitation programs, which have gained increasing attention due to their flexibility and ability to overcome logistical barriers such as distance, transportation difficulties, and mobility limitations. Digital health technologies enable remote monitoring, personalized coaching, and real-time feedback, facilitating patient engagement and self-management [44]. Recent systematic reviews have shown that telerehabilitation provides similar improvements in exercise capacity, risk factor control, and health-related quality of life as traditional center-based programs, while also potentially increasing adherence and reducing costs [44,45]. For patients post-CABG, the availability of various models—ranging from fully home-based programs to hybrid approaches combining occasional in-person visits with remote follow-up—allows tailoring rehabilitation to individual needs, preferences, and risk profiles [45]. This personalization of care is crucial for optimizing long-term outcomes and minimizing the risk of secondary cardiovascular events.

Beyond conventional exercise-based rehabilitation, integrative approaches such as yoga-based cardiac rehabilitation have demonstrated promising long-term benefits. A landmark 15-year legacy randomized controlled trial highlighted a significant reduction in major adverse cardiovascular events (a composite of cardiovascular death, nonfatal myocardial infarction, or stroke) and mortality in post-CABG patients who participated in yoga-based rehabilitation compared to controls [46]. These results underscore the importance of addressing psychosocial and stress-related components of cardiovascular risk alongside traditional physical training, thereby enhancing overall prognosis. Home-based cardiac rehabilitation has also shown superiority or equivalence in several key clinical outcomes compared to center-based programs. Notably, a large comparative study involving cardiovascular patients demonstrated that home-based rehabilitation participants achieved better blood pressure control, improved LDL cholesterol levels, and more effective weight management, without increasing hospital readmissions or medication non-adherence [47]. Such data reinforce the role of decentralized rehabilitation in long-term care pathways, especially for those with limited access to specialized facilities or those balancing rehabilitation with work and family responsibilities.

Regular clinical assessment during long-term follow-up remains indispensable. Outpatient visits should include thorough evaluation of symptoms, functional status, and adherence to prescribed therapies. Cardiovascular imaging, particularly transthoracic echocardiography, is a vital tool to monitor cardiac function and assess possible complications such as ventricular dysfunction. Although imaging frequency should be individualized, routine echocardiographic assessments at 6 to 12 months post-surgery and thereafter based on clinical indications are recommended to guide therapy adjustments and detect early deterioration.

Optimized pharmacological treatment constitutes another cornerstone of long-term management. Guideline-directed medical therapy helps reach and sustain risk factor treatment targets. Indeed, Guidelines emphasize aggressive lipid-lowering strategies, advocating LDL cholesterol targets below 55 mg/dL for very high-risk patients, and even below 40 mg/dL for selected individuals with recurrent events [48]. Similarly, blood pressure control should aim for values under 130/80 mmHg, promoting therapeutic strategies to increase adherence [49,50], and glycemic targets for patients with diabetes should maintain HbA1c levels below 7%, avoiding the development of complications [51,52]. Weight management with a target BMI of 18.5–25 kg/m^2^ further reduces cardiovascular risk. Frequent monitoring and therapeutic adjustments based on these targets improve long-term outcomes and reduce the incidence of graft failure and recurrent ischemia.

Psychosocial factors, including depression, anxiety, and cognitive impairment, significantly affect rehabilitation adherence and cardiovascular prognosis. Studies have highlighted the high prevalence of mental health disorders among post-CABG patients, which often remain underdiagnosed and undertreated. Incorporating routine psychosocial screening and providing multidisciplinary support—comprising psychological counseling, cognitive behavioral therapy, and peer support groups—can enhance patient motivation, reduce emotional distress, and promote healthier lifestyle changes. The integration of mental health care into CR programs is thus an essential component of comprehensive long-term follow-up.

Mobile health (mHealth) technologies represent an emerging adjunct that can enhance self-management and continuous monitoring. Smartphone applications, wearable sensors, and other digital tools facilitate tracking of physical activity, heart rate, blood pressure, and medication adherence in real time. These platforms can provide motivational feedback, reminders, and tailored educational content, all of which have been shown to increase patient engagement and improve clinical parameters. However, successful implementation requires addressing barriers such as digital literacy, patient privacy concerns, and equitable access to technology [45,47].

Despite these advances, significant gaps remain in the evidence base and implementation of long-term follow-up strategies after CABG. Many studies on telerehabilitation and home-based programs have included mixed populations of cardiac patients, limiting specific data on post-CABG cohorts. Furthermore, standardized protocols defining optimal follow-up frequency, intensity of exercise, and multidisciplinary team composition are lacking. Economic evaluations on the sustainability and cost-effectiveness of digital and home-based interventions over prolonged periods are still needed. Lastly, integration of long-term follow-up pathways into routine clinical practice requires overcoming logistical and systemic barriers, including coordination between cardiologists, rehabilitation specialists, primary care providers, and patients themselves.

In conclusion, comprehensive long-term follow-up after CABG requires a multifaceted approach that includes continued rehabilitation through phase III programs, close clinical and imaging surveillance, strict adherence to pharmacological and lifestyle interventions, and attention to psychosocial health. The incorporation of home-based and telehealth modalities enhances accessibility and adherence, while mHealth tools offer promising opportunities for patient-centered care. Achieving guideline-recommended targets for LDL cholesterol, blood pressure, glycemic control, and BMI remains fundamental to reducing secondary cardiovascular risk (Table 2). Future research should focus on refining individualized follow-up strategies, evaluating long-term cost-effectiveness, and addressing disparities in care access to optimize outcomes for patients after CABG.

## 5. Tailored Management of Patients After CABG in Clinical Practice

**Figure 1:**
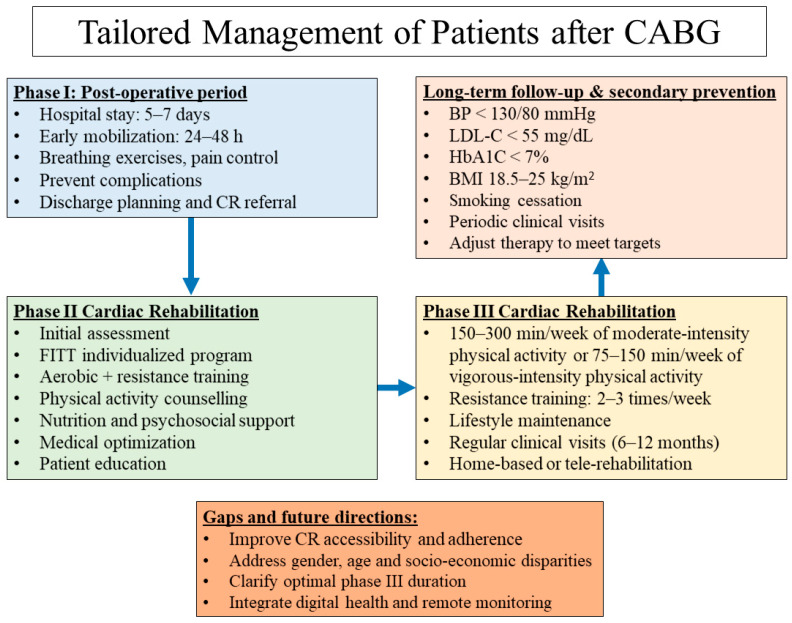
Management of patients after CABG in clinical practice: from the post-operative period to long-term follow-up. BMI, body mass index; BP, blood pressure; CABG, coronary artery bypass grafting; CR, cardiac rehabilitation; FITT, frequency, intensity, time, and type of exercise; HbA1C, glycated haemoglobin; LDL-C, low-density lipoprotein cholesterol.

The optimal care of patients following CABG requires an individualized, stepwise approach that integrates evidence-based medical management, structured cardiac rehabilitation, and long-term secondary prevention. A clearly defined care pathway, starting from the immediate postoperative period and extending through phases I, II, and III of cardiac rehabilitation, ensures continuity of care, maximizes recovery, and reduces recurrent cardiovascular events (Figure 1).

In the postoperative in-hospital period (Phase I), the main goals are early mobilization, prevention of complications (e.g., pulmonary infections, thromboembolism), pain control, psychological support, and discharge planning. Mobilization typically begins 24–48 h after surgery, progressing gradually with assistance from physiotherapists and rehabilitation nurses [53]. Breathing exercises, bed-to-chair transfers, and corridor walking are introduced in a stepwise manner. During this period, patients should receive structured education on medication adherence, incision care, activity limitations (e.g., sternal precautions), and the importance of lifestyle modification. Once the incision is healed, patients may also benefit from brief guidance on basic scar desensitization and mobility under supervision of the rehabilitation team. Prior to discharge, the care team should formally refer the patient to the Phase II cardiac rehabilitation program, as early referral increases participation and outcomes.

Phase II cardiac rehabilitation is delivered in a supervised setting and includes individualized exercise training based on prior functional assessment, nutritional counseling, psychosocial support, smoking cessation strategies, and medication optimization. This phase also allows fine-tuning of cardiovascular risk factor management. According to recent guidelines, LDL-C targets should be <55 mg/dL (1.4 mmol/L) in very high-risk patients, and <40 mg/dL (1.0 mmol/L) in those with recurrent events. For blood pressure, guidelines recommend a target of <130/80 mmHg and, in those with diabetes, HbA1c should be maintained below 7%, though a more lenient goal (e.g., 7–8%) may be appropriate in elderly or frail patients [54]. BMI should be reduced below 25 kg/m^2^, but greater emphasis should be placed on reducing visceral adiposity and improving body composition [55].

After completing the structured program, patients transition to Phase III cardiac rehabilitation, which represents the long-term maintenance and self-management phase. This phase focuses on maintaining exercise routines, sustained adherence to lifestyle modifications, and regular medical follow-up. Home-based or community-based exercise programs, possibly supported by digital health tools, have shown effectiveness in maintaining gains from Phase II, especially in patients with limited access to center-based programs [43].

In terms of long-term follow-up, a structured schedule is essential. Clinical visits are typically scheduled at 3–6 months post-rehabilitation and then annually. Each visit should include history-taking, symptom review, vital signs, and a thorough medication review [56]. Laboratory tests (lipid panel, HbA1c, renal function) should be performed periodically, usually every 6–12 months. Risk factor optimization must be approached dynamically. Echocardiography is recommended at baseline and repeated if there is a change in symptoms or in patients with reduced ejection fraction. In high-risk individuals (e.g., multiple grafts, incomplete revascularization), periodic non-invasive ischemia testing (e.g., stress echocardiography, myocardial perfusion imaging, or coronary CT angiography) may be considered to assess graft patency and guide further management.

Despite robust evidence supporting this pathway, gaps remain in implementation and research. Global participation in cardiac rehabilitation remains low (<50% in many countries), with disparities in referral, access, and adherence—particularly among women, older adults, and socioeconomically disadvantaged populations [57]. Moreover, the optimal duration and format of Phase III CR are not well established. The integration of digital health, remote monitoring, and individualized behavioral strategies represents a promising direction for future research. Large, pragmatic trials are needed to assess the effectiveness of hybrid models and to refine long-term follow-up algorithms beyond five years post-CABG [58]. Therefore, tailored management after CABG should follow a structured, phase-based model integrating clinical, behavioral, and rehabilitative strategies. It should be aligned with current guideline targets for risk factor control, supported by a multidisciplinary team, and adapted to patient-specific needs and preferences. Future efforts must focus on increasing equitable access to cardiac rehabilitation, improving long-term adherence, and generating high-quality evidence to personalize follow-up beyond the first postoperative years.

## 6. Conclusions

CABG remains a cornerstone intervention in the management of advanced CAD. However, optimal outcomes depend not only on surgical success but also on structured, long-term care strategies. Cardiac rehabilitation—spanning Phases I through III—offers a comprehensive, evidence-based framework for physical recovery, risk factor control, psychosocial support, and secondary prevention. Initial assessments, especially for exercise prescription using the FITT model, are essential to ensure patient safety and efficacy of rehabilitation. Individualized exercise programs, combined with aggressive risk factor management, significantly reduce recurrent events and mortality. The integration of multidisciplinary care, early mobilization, and long-term follow-up supports a holistic, patient-centered recovery. Nevertheless, gaps remain—particularly in global access to CR, participation disparities, and lack of long-term data beyond five years post-CABG. Future efforts must prioritize wider implementation, personalization through digital health tools, new evidence and high-quality research on the short- and long-term adherence and outcomes. In clinical practice, CABG care must go beyond surgery and encompass a continuum of care through rehabilitation and secondary prevention—turning an invasive and stressful intervention into a lifelong opportunity for cardiovascular risk reduction and improved quality of life, based on a multidisciplinary approach and tailored management.

## Figures and Tables

**Table 2:** Key targets and components of Long-Term Follow-Up after CABG.

Component	Target/Recommendation
LDL-C	<55 mg/dL in very high-risk patients; <40 mg/dL in selected very high-risk patients (extreme risk)
Blood pressure	<130/80 mmHg
Glycemic control (HbA1C)	<7%
BMI	18.5–25 kg/m^2^
Physical activity	150–300 min per week of moderate-intensity physical activity, or 75–150 min of vigorous-intensity physical activity + training twice weekly
Cardiac Imaging	Echocardiography at 6–12 months post-CABG, then as clinically indicated
Rehabilitation Modality	Phase III home-based, telerehabilitation, or hybrid models
Psychosocial Support	Routine screening and management of depression, anxiety, cognitive impairment
Medical Therapy Adherence	Periodic review and use of mHealth tools to enhance compliance

BMI, body mass index; CABG, coronary artery bypass grafting; HbA1C, glycated haemoglobin; LDL-C, low-density lipoprotein cholesterol; mHealth, mobile health.

## Data Availability

No new date were created or analyzed in this study. Data sharing is not applicable to this article.
